# Knockdown of miR-155 protects microglia against LPS-induced inflammatory injury via targeting RACK1: a novel research for intracranial infection

**DOI:** 10.1186/s12950-017-0162-7

**Published:** 2017-08-09

**Authors:** Haiyan Yin, Shuwen Song, Xudong Pan

**Affiliations:** 1 Department of Neurology, Affiliated Hospital of Jining Medical University, Jining, 272000 China; 2 Department of Neurology, Affiliated Hospital of Jining Medical University, Jining, 272000 China; 3 Department of Infectious Diseases, Jining No. 1 People’s Hospital, Jining, 272000 China; 4 Department of Neurology, The Affiliated Hospital of Qingdao University, No. 16, Jiangsu Road, Qingdao, 266000 China

**Keywords:** Intracranial inflammation, miR-155, Inflammation, Cell injury, Microglia, RACK1

## Abstract

**Background:**

Intracranial infection, one of the complications of traumatic brain injury, is usually associated with inflammation. Several microRNAs (miRNAs), including miR-155, have been reported to be critical modulators in peripheral and central nervous system inflammation. In this study, we investigated the role of miR-155 in lipopolysaccharide (LPS)-induced inflammatory injury in mouse microglia BV2 cells.

**Results:**

The expression level of miR-155 was significantly up-regulated after LPS stimulation in BV2 cells. LPS administration decreased BV2 cell viability, promoted apoptosis and increased the release of pro-inflammatory cytokines; while miR-155 knockdown rescued BV2 cell from LPS-induced injury. RACK1 was a directly target of miR-155. Interestingly, miR-155 knockdown did not attenuate LPS-induced inflammatory injury when RACK1 was knocked down. The mechanistic study indicated that miR-155 knockdown deactivated MAPK/NF-κB and mTOR signaling pathways under LPS-treated conditions.

**Conclusions:**

Knockdown of miR-155 protected mouse microglia BV2 cells from LPS-induced inflammatory injury via targeting RACK1 and deactivating MAPK/NF-κB and mTOR signaling pathways.

## Background

Intracranial infection, one of the complications of traumatic brain injury, is relatively uncommon after brain injury, but it is associated with poor neurologic prognosis and cognitive disorder [1]. Post traumatic intracranial infection is generally due to contamination that caused by foreign body entered into the brain parenchyma after the injury [2]. Intracranial infection is commonly manifested as seizures, altered mental status, and cranial nerve palsy [3]. Inflammation is very common after intracranial infection [4]. Therapeutic management strategies for intracranial infection mainly include antibiotics therapy and surgical strategy. Intracranial infection is usually proceed rapidly and results in significant morbidity and mortality if appropriate therapies are not initiated in time [3]. Intracranial infection-related mortality rate was reported to be as high as 28% [5]. Therefore, development of effective targeted pharmacological strategies would be beneficial to such patient population with intracranial infection.

MicroRNAs (miRNAs) are small non-coding RNAs which regulate the expression of protein-encoding genes at the post-transcriptional level. MiRNAs are involved in various cellular processes such as cell proliferation, apoptosis, and differentiation [6–8]. Additionally, miRNAs also affect the fat and cholesterol metabolism, nerve development, hormone secretion, and immune response [9, 10]. MiRNAs are abundant in brain tissues and play a variety of regulatory functions in the central nervous system (CNS) [11]. It has been reported that miRNAs played key roles in the process of nerve development and injury repair [8, 12, 13]. Recent studies suggested that miRNA expression was dysregulated in psychiatric and neurological disorders sufferers [14–16].

MiRNA-155 (miR-155) is derived from the non-coding transcript of the proto-oncogene B-cell integration cluster (*bic*) [17]. MiR-155 plays essential roles in autoimmune diseases and inflammatory responses [18]. In several in vitro and in vivo studies, silencing of miR-155 expression ameliorated the deterioration of the disease and delayed the autoimmune encephalomyelitis and rheumatoid arthritis [19–22]. Another in vivo study had demonstrated that deficiency of miR-155 ameliorated autoimmune inflammation of systemic lupus erythematosus in mice [18]. Therefore, miR-155 might be a pro-inflammatory miRNA in inflammatory diseases and silencing of its expression might prevent inflammation. It has been reported that the expression of miR-155 is altered in murine glial cells administrated with the bacterial endotoxin lipopolysaccharide (LPS) [23]. However, the role of miR-155 in intracranial infection is still unclear. Thus, in the present study, we investigated the functional role of miR-155 in intracranial infection in microglia cell injury which was induced by LPS.

## Methods

### Cell culture and LPS treatment

Mouse microglial BV2 cell line was cultured in high-glucose Dulbecco’s modified Eagle’s medium (DMEM) supplemented with 10% (*v*/v) fetal bovine serum (FBS; catalog #26140; Gibco, Waltham, MA), 100 IU/ml penicillin, and 10 μg/ml streptomycin (catalog #15140; Invitrogen, Waltham, MA). Cells were maintained in a humidified incubator with 95% air and 5% CO_2_ atmosphere at 37 °C. Culture medium containing the appropriate agents was replaced every other day.

For LPS treatment, BV2 cells were cultured in culture medium containing 1% FBS. Inflammation was induced by LPS which was from *E. coli* O111:B4 (L4391, Sigma-Aldrich). Cells were treated with 10 μg/ml LPS for 2 h, 4 h and 5 h; or were treated with different concentrations of LPS (0, 1, 5, 10, and 20 μg/ml) for 5 h.

### Cell Counting Kit-8 (CCK-8) assay

Cells were seeded in 96-well plate at a density of 5 × 10^3^ cells/well. Cell viability was assessed by using the CCK-8 kit (Dojindo Molecular Technologies, Gaithersburg, MD). Briefly, after LPS stimulation, CCK-8 solution was added to the culture medium, and the cultures were incubated for 1 h at 37 °C in humidified 95% air and 5% CO_2_. The absorbance was measured at 450 nm by Microplate Reader (Bio-Rad, Hercules, CA).

### Apoptosis assay

Flow cytometry analysis was performed to identify and quantify the apoptotic cells by using the Annexin V-FITC/PI apoptosis detection kit (Beijing Biosea Biotechnology, Beijing, China). Cells (1 × 10^5^ cells/well) were seeded in 6 well-plate. After LPS stimulation, cells were washed twice with cold phosphate buffered saline (PBS) and were re-suspended in 200 μl binding buffer containing 10 μl FITC–annexin V and 5 μl PI. After incubation in the dark at room temperature for 15 min, a sample of 300 μl binding buffer was added, the cells were then immediately analyzed by the flow cytometer (Beckman Coulter, USA).

### Enzyme-linked immunosorbent assay (ELISA)

Cell culture supernatant was collected from 24-well plates after LPS administration, and then the concentrations of inflammatory cytokines were measured by ELISA according to the manufacturer’s protocols (R&D Systems, Abingdon, UK) and normalized to cell protein concentrations.

### Cell transfection

MiR-155 mimic, miR-155 inhibitor, RACK1 targeted siRNA and their correspondingly negative controls (NC) were all synthesized by GenePharma Co. (Shanghai, China). Cell transfections were performed by using Lipofectamine 3000 reagent (Invitrogen) following the manufacturer’s protocol.

### Quantitative reverse transcription polymerase chain reaction (qRT-PCR)

Total RNA was extracted from cells after LPS administration by using Trizol reagent (Life Technologies Corporation, Carlsbad, CA, USA) according to the manufacturer’s instructions. Taqman MicroRNA Reverse Transcription Kit and Taqman Universal Master Mix II with TaqMan MicroRNA Assay (Applied Biosystems, Foster City, CA, USA) were used for testing the expression level of miR-155 and mRNAs expressions in cells.

### Dual luciferase activity assay

The potential target sequence was predicted, and generated by PCR. Luciferase reporter was constructed by inserting the RACK1 3’UTR carrying the putative miR-155-binding sites into pMiR-report vector. The reporter construct (RACK1 promotor) or control vector (U6) were co-transfected into cells with miR-155 mimic or miRNA scramble by using Lipofectamine 3000 (Life Technologies, USA). Reporter assays were done by the dual-luciferase assay system (Promega) following to the manufacturer’s information.

### Western blot analysis

The proteins used for western blotting assay were extracted by using radioimmunoassay (RIA) lysis buffer (Beyotime Biotechnology, Shanghai, China) supplemented with protease inhibitors (Roche, Guangzhou, China). The protein samples were quantified by the BCA™ Protein Assay Kit (Pierce, Appleton, WI, USA). Western blot system was established by using a Bio-Rad Bis-Tris Gel system according to the manufacturer’s instructions. Glyceraldehyde-3-phosphate dehydrogenase (GAPDH) antibody was purchased from Sigma. Primary antibodies were prepared in 5% blocking buffer at a dilution of 1:1000, then been incubated with the polyvinylidene difluoride (PVDF) membranes at 4 °C overnight, followed by wash and incubation with secondary antibody marked by horseradish peroxidase for 1 h at room temperature. After rinsing, the membranes carried blots and antibodies were transferred into Bio-Rad ChemiDoc™ XRS system, and then 200 μl Immobilon Western Chemiluminescent HRP Substrate (Millipore, MA, USA) was added to cover the membrane surface. The signals were captured and the intensity of the bands was quantified using Image Lab™ Software (Bio-Rad, Shanghai, China).

### Statistical analysis

All experiments were repeated at least three times. The results of multiple experiments are presented as mean ± standard deviation (SD). Statistical analyses were performed by using SPSS 19.0 statistical software. *P*-values were calculated using one-way analysis of variance for more than two groups, or two-tailed Student’s t test between two groups. *P*-value of <0.05 was considered to indicate a statistically significant result.

## Results

### LPS induced inflammatory injury in mouse microglia BV2 cells

**Fig. 1:**
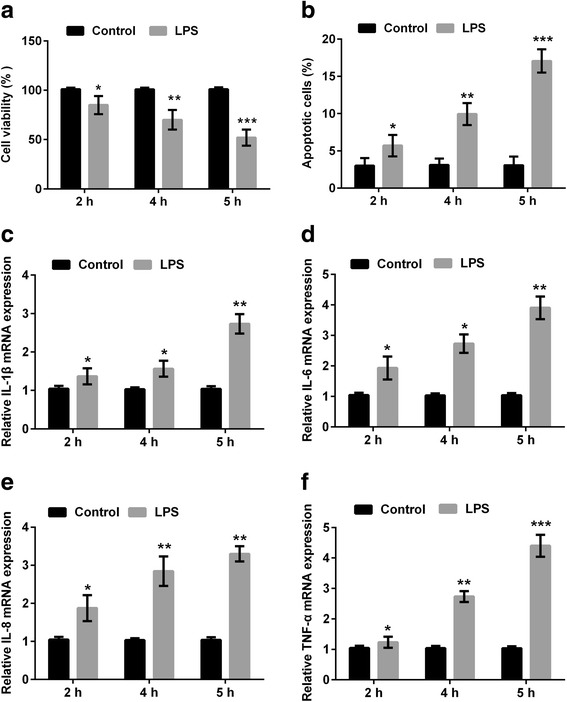
LPS damaged mouse microglia BV2 cells in a time-dependent manner. BV2 cells were pre-treated with 10 μg/ml LPS for 0, 2, 4 and 5 h. Cells without LPS treatment were used as control. **a** Cell viability was measured by CCK-8 assay. **b** Cell apoptosis was measured by flow cytometry. **c**-**f** Relative mRNA expressions of IL-1β, IL-6, IL-8, and TNF-α in LPS-treated cells and control cells were measured by quantitative RT-PCR. *n* = 3. CCK-8: Cell Counting Kit-8; GAPDH: glyceraldehyde-3-phosphate dehydrogenase; IL: interleukin; LPS: lipopolysaccharide; RT-PCR: reverse transcription polymerase chain reaction; TNF-α: tumor necrosis factor alpha. *P < 0.05, **P < 0.01, *** *P* < 0.001

Initially, BV2 cells were subjected to 10 μg/ml LPS for various times (2, 4, and 5 h), and then cell viability, apoptosis and the release of inflammatory cytokines were detected. As results shown in Fig. 1a-f, cell viability was significantly reduced (*P* < 0.05, *P* < 0.01, or *P* < 0.001), while apoptotic cell rate and the expressions of inflammatory cytokines (IL-1β, IL-6, IL-8 and TNFα) were all significantly increased (*P* < 0.05, *P* < 0.01, or *P* < 0.001) in response to 2, 4, and 5 h exposure of 10 μg/ml LPS. These data suggested that LPS-induced inflammatory injury in BV2 cells in a time-dependent manner, and 5 h was selected as one LPS-stimulating condition for use in the following experiments.

**Fig. 2:**
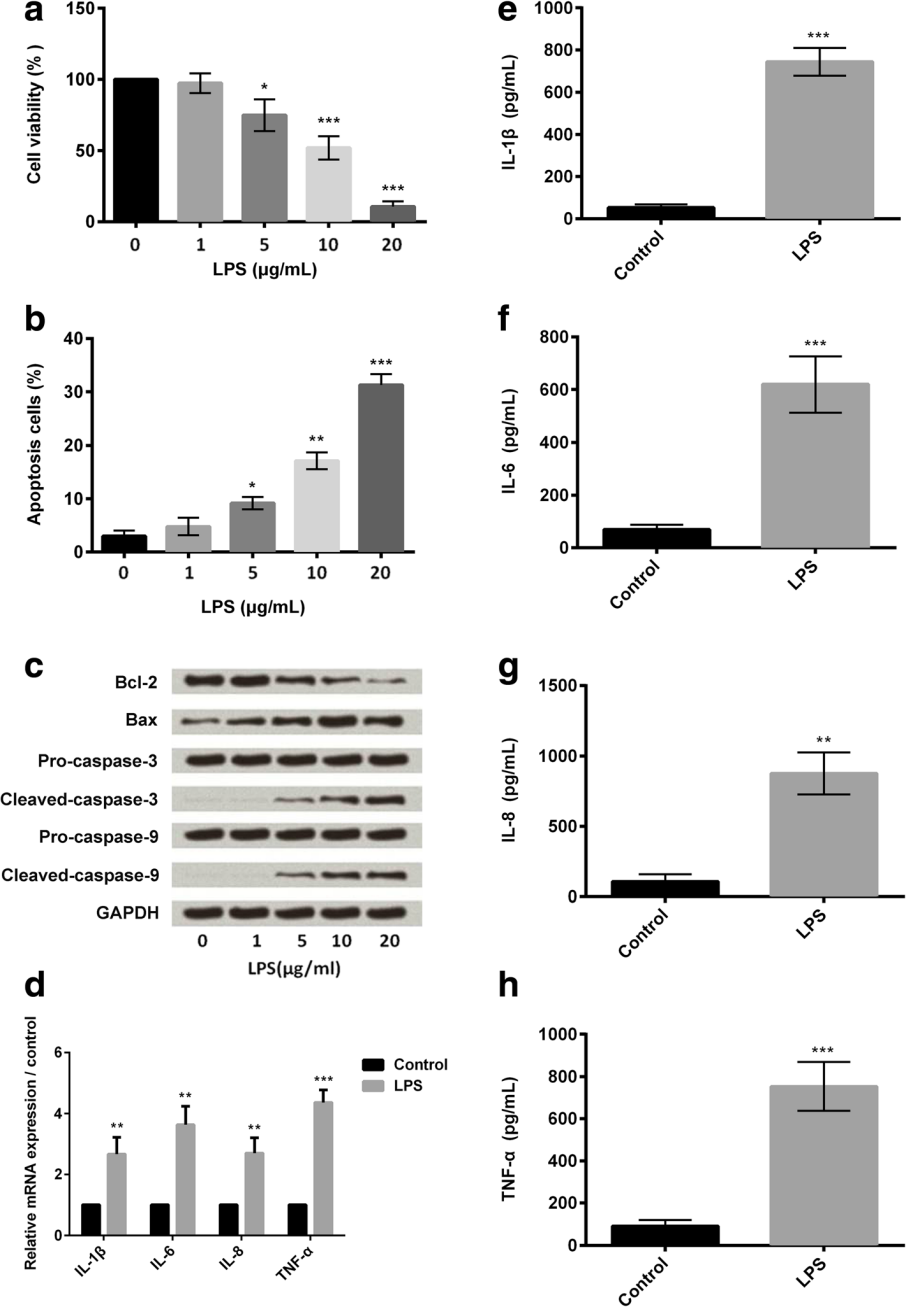
LPS damaged mouse microglia BV2 cells in a dose-dependent manner. BV2 cells were pre-treated with different concentrations of LPS (0, 1, 5, 10, and 20 μg/ml) for 5 h. Cells without LPS treatment were used as control. **a** Cell viability was measured by CCK-8 assay (*n* = 3). **b** Cell apoptosis was measured by flow cytometry (*n* = 3). **c** Expression of anti-apoptotic (Bcl-2) and pro-apoptotic (Bax, caspase-3, and caspase-9) proteins were measured by western blot analysis. **d** Relative mRNA expressions of IL-1β, IL-6, IL-8, and TNF-α in LPS-treated cells and control cells were measured by quantitative RT-PCR (*n* = 3). (**e**-**h**) Concentrations of IL-1β, IL-6, IL-8, and TNF-α in LPS-treated cells and control cells were measured by ELISA (*n* = 3). CCK-8: Cell Counting Kit-8; GAPDH: glyceraldehyde-3-phosphate dehydrogenase; ELISA: enzyme-linked immunosorbent assay; IL: interleukin; LPS: lipopolysaccharide; RT-PCR: reverse transcription polymerase chain reaction; TNF-α: tumor necrosis factor alpha. *P < 0.05, **P < 0.01, *** *P* < 0.001

Meanwhile, BV2 cells were pre-treated with different concentrations of LPS (0, 1, 5, 10, and 20 μg/ml) for 5 h, and cell viability and apoptosis were evaluated. Data in Fig. 2a-b showed that, BV2 cells treated with 5, 10, and 20 μg/ml LPS resulted in a significant decreased in cell viability, and a significance increase in apoptotic cell rate (*P* < 0.05, *P* < 0.01, or *P* < 0.001). Besides, 5, 10, and 20 μg/ml LPS administration decreased the expression of anti-apoptotic protein (Bcl-2) and increased the expressions of pro-apoptotic proteins (Bax, caspase-3, and caspase-9) (Fig. 2c). It seems that LPS induced cell damage in BV2 cells also in a dose-dependent manner, since a higher concentration of LPS resulted in a lower viability of BV2 cells and a higher apoptotic cell rate. Concentration of 10 μg/ml was selected as another LPS-stimulating condition for use in the later study.

Next, we detected the effect of 10 μg/ml LPS for a culture time of 5 h on the release of pro-inflammatory cytokines. qRT-PCR analytical results showed that the relative mRNA expression levels of IL-1β, IL-6, IL-8, and TNF-α were increased markedly in the LPS-treated cells compared to the control cells (*P* < 0.01 or *P* < 0.001) (Fig. 2d). Likewise, ELISA results showed the increased concentrations of these inflammatory cytokines in the LPS-injured cells (*P* < 0.01 or *P* < 0.001; Fig. 2e-h). These findings combined with the previous results suggested that LPS induced inflammatory injury of mouse microglia BV2 cells in vitro.

### LPS increased miR-155 expression during cell injury

**Fig. 3:**
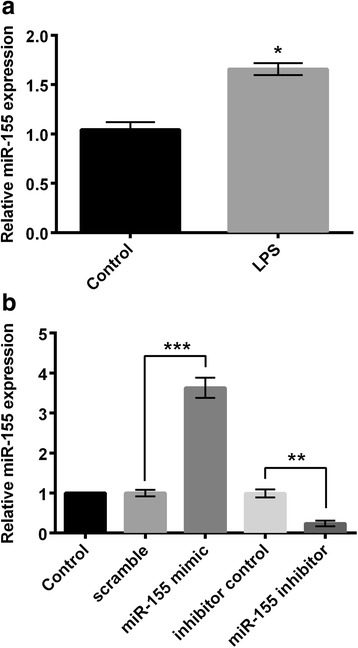
MiR-155 was upregulated in LPS-injured BV2 cells. BV2 cells were treated with 10 μg/ml LPS for 5 h. Quantitative RT-PCR was used to measure relative expression of miR-155. **a** Expression of miR-155 in LPS-treated cells and control cells; **b** Expression of miR-155 in cells transfected with miR-155 mimic, miR-155 inhibitor, or their correspondingly negative controls (scramble and inhibitor control). *n* = 3. LPS: lipopolysaccharide; RT-PCR: reverse transcription polymerase chain reaction. *P < 0.05, **P < 0.01, ***P < 0.001

We then measured the effect of LPS on miR-155 expression by qRT-PCR. The results showed that the relative expression of miR-155 was significantly increased in the LPS-treated cells compared to the control cells (without LPS treatment) (*P* < 0.05, Fig. 3a). These data hint us that miR-155 might play a role in LPS-induced cell damage in BV2 cells. Thus, we transfected miR-155 mimic, miR-155 inhibitor, and their correspondingly controls (scramble and inhibitor control) into BV2 cells to alter miR-155 expressions and detect whether miR-155 was involved in LPS-induced cell damage. As shown in Fig. 3b, miR-155 mimic significantly promoted miR-155 expression (*P* < 0.001) when compared to scramble group, and miR-155 inhibitor transfection significantly decreased miR-155 expression when compared to inhibitor control (*P* < 0.01).

### Down-regulation of miR-155 rescued BV2 cells from LPS-induced inflammatory injury

**Fig. 4:**
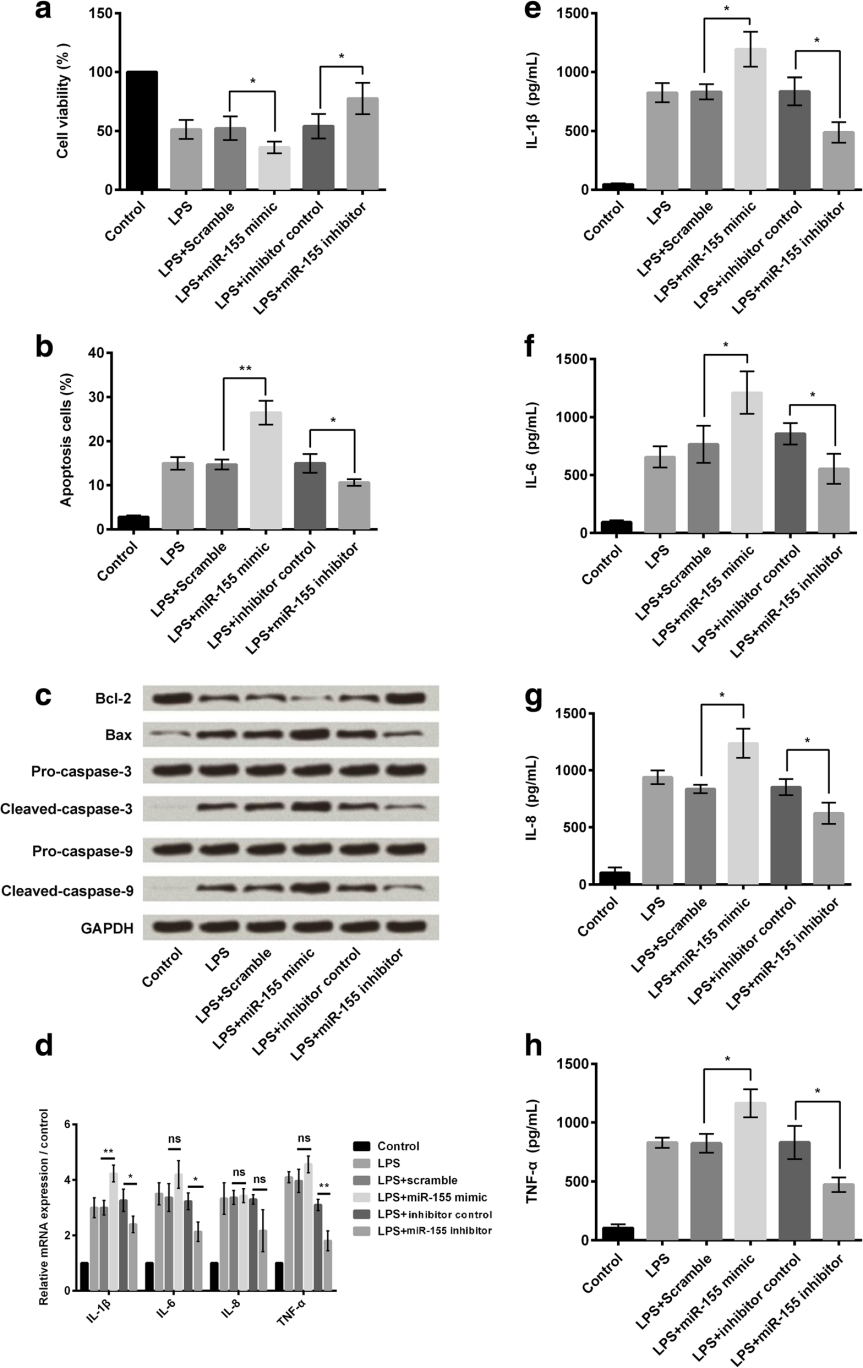
Down-regulation of miR-155 inhibited LPS-induced cell injury. BV2 cells were administrated with LPS, LPS + scramble, LPS + miR-155 mimic, LPS+ inhibitor control, and LPS + miR-155 inhibitor. **a** Cell viability was measured by CCK-8 assay (*n* = 3). **b** Cell apoptosis was measured by flow cytometry (*n* = 3). **c** Expression of anti-apoptotic (Bcl-2) and pro-apoptotic (Bax, caspase-3, and caspase-9) proteins were measured by western blot analysis. **d** Relative mRNA expressions of IL-1β, IL-6, IL-8, and TNF-α were measured by quantitative RT-PCR (*n* = 3). **e-h** Concentrations of IL-1β, IL-6, IL-8, and TNF-α were measured by ELISA (*n* = 3). CCK-8: Cell Counting Kit-8; GAPDH: glyceraldehyde-3-phosphate dehydrogenase; ELISA: enzyme-linked immunosorbent assay; IL: interleukin; LPS: lipopolysaccharide; RT-PCR: reverse transcription polymerase chain reaction; TNF-α: tumor necrosis factor alpha. ns, no significance, *P < 0.05, **P < 0.01

To assess the effect of miR-155 on LPS-induced cell injury, BV2 cells were treated with LPS, LPS + miR-155 mimic, and LPS + miR-155 inhibitor, and then cell viability and apoptosis were analyzed. CCK-8 assay results (Fig. 4a) showed that up-regulation of miR-155 significantly decreased cell viability of LPS-treated cells (*P* < 0.05), whereas down-regulation of miR-155 increased cell viability of these cells (*P* < 0.05). Flow cytometry detection results (Fig. 4b) showed that up-regulation of miR-155 significantly increased apoptosis of LPS-treated cells (*P* < 0.01), whereas down-regulation of miR-155 decreased apoptosis (*P* < 0.05). Western blot analytical results showed that up-regulation of miR-155 reduced Bcl-2 protein expression level while increased Bax, cleaved caspase-3 and cleaved caspase-9 protein expression levels; down-regulation of miR-155 affected these protein expressions resulted in the contrary results (Fig. 4c).

Effect of miR-155 on pro-inflammatory cytokines was determined by qRT-PCR and ELISA. qRT-PCR analytical results showed that up-regulation of miR-155 increased the relative mRNA expression levels of IL-1β, IL-6, IL-8, and TNF-α in the LPS-injured cells, whereas down-regulation of miR-155 showed opposite results (*P* < 0.05 or *P* < 0.01, Fig. 4d). ELISA results showed increased concentrations of these inflammatory cytokines in miR-155 mimic transfected cells and decreased concentrations in miR-155 inhibitor transfected cells (*P* < 0.05, Fig. 4E-H). These findings suggested that up-regulation of miR-155 increased LPS-induced cell injury, whereas down-regulation of miR-155 expression alleviated it.

### RACK1 was a target of miR-155

**Fig. 5:**
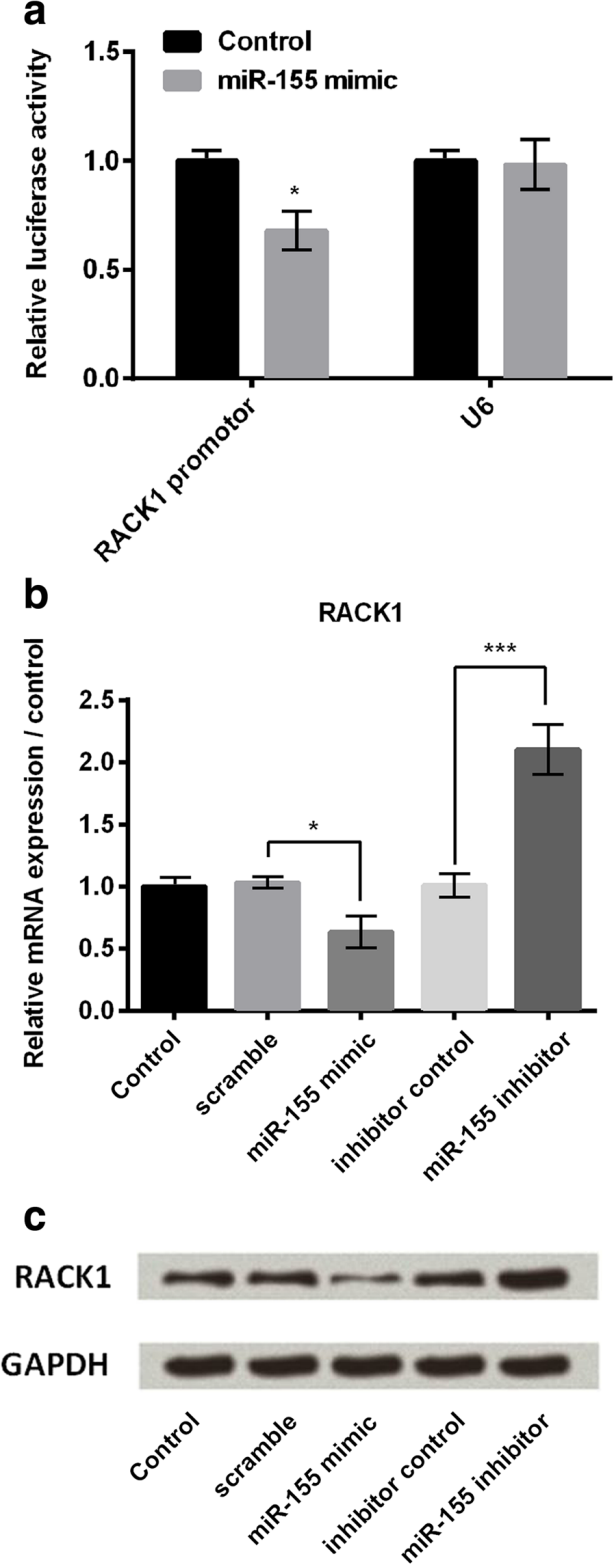
RACK1 was a directly target of miR-155. **a** Relative luciferase activity of recombinant RACK1 promotor and U6 (control) vectors in BV2 cells which were co-transfected with scramble control or miR-155 mimic (*n* = 3). **b** Quantitative RT-PCR was performed to measure relative mRNA expression of RACK1 (*n* = 3), and (**c**) Western blot analysis was performed to measure the protein expressions of RACK1 in cells which were transfected with scramble, miR-155 mimic, inhibitor control, and miR-155 inhibitor. GAPDH: glyceraldehyde-3-phosphate dehydrogenase; RT-PCR: reverse transcription polymerase chain reaction. *P < 0.05, ***P < 0.001

Dual luciferase reporter assay was performed to investigate the target of miR-155. Cells were co-transfected with the recombinant reporter vector and miR-155 mimic by using Lipofectamine 3000. Results showed that luciferase activity was significantly decreased in the cells transfected with recombinant reporter vector containing RACK1 promoter and miR-155 mimic, as compared to the negative control group (*P* < 0.05, Fig. 5a), suggesting miR-155 directly targeted 3’UTR of RACK1, and RACK1 might be a directly target of miR-155.

To further confirm that RACK1 was a target of miR-155, we measured the relative mRNA expression of RACK1 in cells after been transfected with miR-155 mimic, miR-155 inhibitor or their correspondingly controls. Figure 5b shows that miR-155 mimic transfection significantly decreased the mRNA expression level of RACK1 when compared with scramble control group (*P* < 0.05), while miR-155 inhibitor transfection increased the mRNA expression of RACK1 when compared with inhibitor control (*P* < 0.001). Western blot analytical results showed similar results about RACK1 protein expressions (Fig. 5c). These results suggested that RACK1 might be a directly target of miR-155, and the expression of RACK1 was negatively regulated by miR-155.

### Down-regulation of miR-155 rescued BV2 cells from LPS-induced inflammatory injury via modulating RACK1

**Fig. 6:**
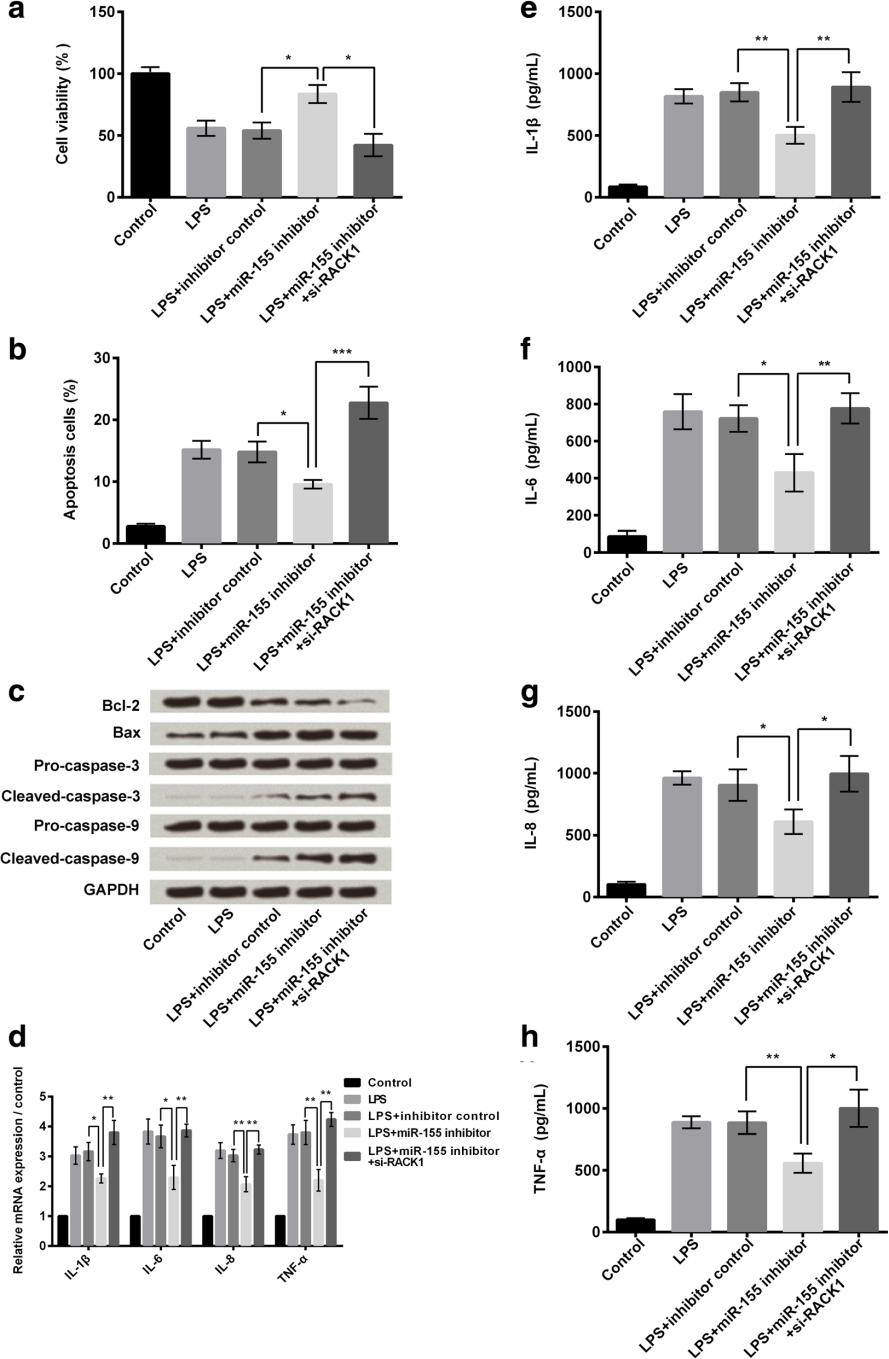
RACK1 knockdown promoted LPS-induced cell injury. BV2 cells were treated with LPS, LPS+ inhibitor control, LPS + miR-155 inhibitor, and LPS+ miR-155 inhibitor + si-RACK1. **a** Cell viability was measured by CCK-8 assay (*n* = 3). **b** Cell apoptosis was measured by flow cytometry (*n* = 3). **c** Expression of anti-apoptotic (Bcl-2) and pro-apoptotic (Bax, caspase-3, and caspase-9) proteins were measured by western blot analysis. **d** Relative mRNA expressions of IL-1β, IL-6, IL-8, and TNF-α were measured by quantitative RT-PCR (*n* = 3). **e**-**h** Concentrations of IL-1β, IL-6, IL-8, and TNF-α were measured by ELISA (*n* = 3). CCK-8: Cell Counting Kit-8; GAPDH: glyceraldehyde-3-phosphate dehydrogenase; ELISA: enzyme-linked immunosorbent assay; IL: interleukin; LPS: lipopolysaccharide; RT-PCR: reverse transcription polymerase chain reaction; TNF-α: tumor necrosis factor alpha. *P < 0.05, **P < 0.01, ***P < 0.001

To determine the role of RACK1 in miR-155 modulated BV2 cells which were injured by LPS, the expressions of miR-155 and/or RACK1 were knocked down in BV2 cells. Cell viability and apoptosis of cells which were treated with LPS, LPS + NC, LPS + miR-155 inhibitor, or LPS + miR-155 inhibitor + si-RACK1 were measured. As shown in Fig. 6a and b, after LPS administration, miR-155 inhibitor transfection alone significantly increased cell viability and decreased apoptosis (both *P* < 0.05). However, the down-regulation of miR-155 did not increase cell viability and decrease apoptosis under LPS-treated condition when RACK1 was knocked down (*P* < 0.05 or *P* < 0.001). Western blot analysis about apoptotic related factors expressions suggested the similar results for cell apoptosis (Fig. 6c).

We also evaluated the impact of RACK1 on inflammatory cytokines by qRT-PCR and ELISA. qRT-PCR results showed that transfection of miR-155 inhibitor alone in LPS-induced cells decreased the relative mRNA expression levels of IL-1β, IL-6, IL-8, and TNF-α, while transfection with both miR-155 inhibitor and si-RACK1 increased the expression of these pro-inflammatory cytokines (*P* < 0.05 or *P* < 0.01, Fig. 6d). ELISA test results (Fig. 6e-h) showed that miR-155 inhibitor transfection alone increased the concentrations of these factors, and while both miR-155 inhibitor and si-RACK1 transfection decreased concentrations of IL-1β, IL-6, IL-8, and TNF-α (*P* < 0.05 or *P* < 0.01). These findings suggested that RACK1 knockdown could promote LPS-induced cell injury and inflammation, even if the expression of miR-155 was down-regulated. Therefore, we speculated that down-regulation of miR-155 rescued BV2 cells from LPS-induced inflammatory injury might be via modulating RACK1.

### Down-regulation of miR-155 protected BV2 cells from LPS-induced inflammatory injury via deactivation of MAPK/NF-κB and mTOR pathways

**Fig. 7:**
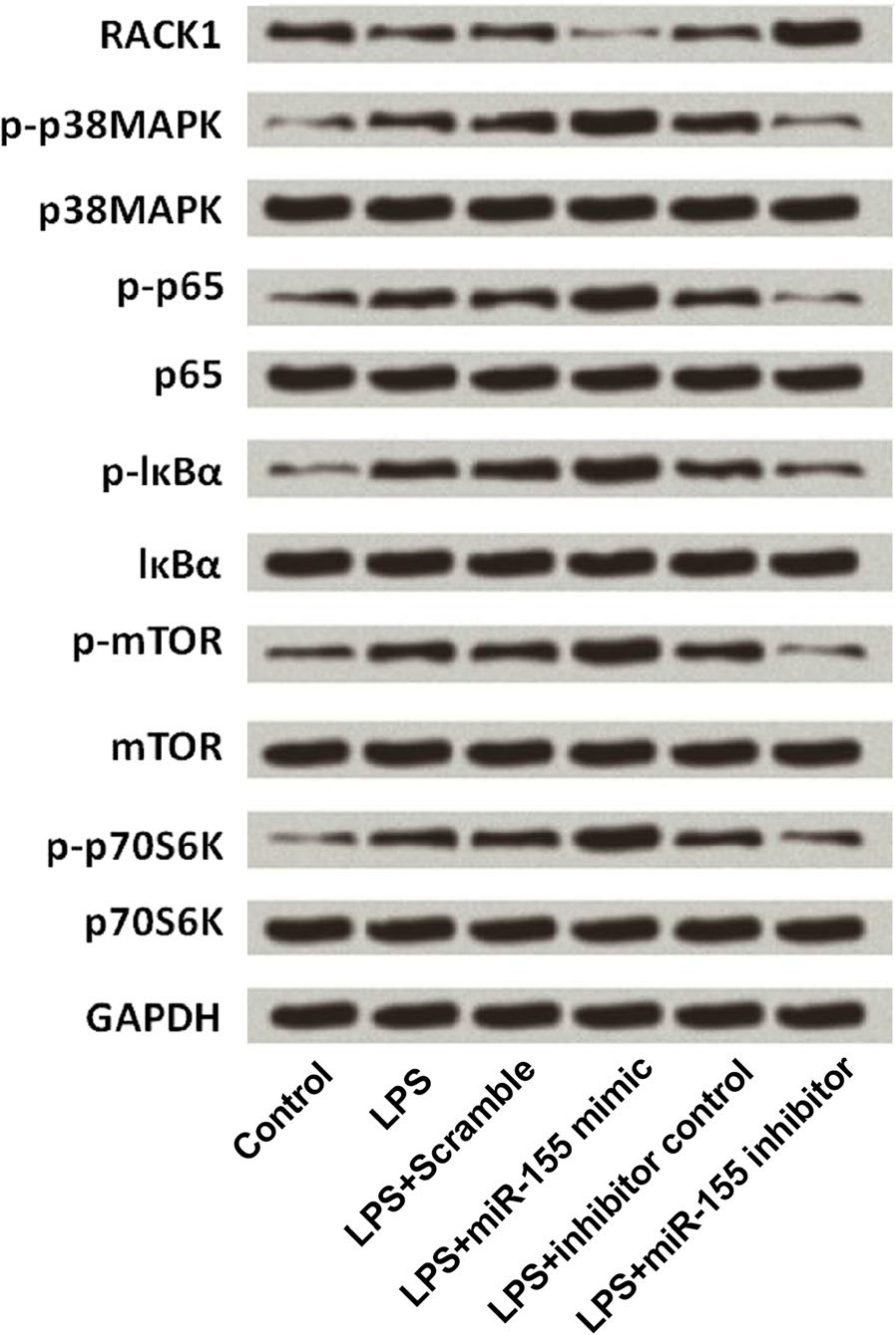
Down-regulation of miR-155 protected BV2 cells from LPS-induced inflammatory injury via deactivation of MAPK/NF-κB and mTOR pathways. Western blot analysis was used to measure the expressions of RACK1, MAPK protein (p38), NF-κB proteins (p65 and lκBα), and mTOR proteins (mTOR and p70S6K) in BV2 cells which were treated with LPS, LPS + scramble, LPS + miR-155 mimic, LPS+ inhibitor control, and LPS + miR-155 inhibitor. GAPDH: glyceraldehyde-3-phosphate dehydrogenase; LPS: lipopolysaccharide; MAPK: mitogen activated protein kinase; NF-κB: nuclear factor kappa B; mTOR: mammalian target of rapamycin

Furthermore, we investigated the underlying mechanisms of which miR-155 down-regulation rescued BV2 cells from LPS-induced inflammatory injury. Western blot analysis was performed and the expressions of mainly factors in mitogen activated protein kinase (MAPK)/nuclear factor kappa-B (NF-κB) and mammalian target of rapamycin (mTOR) signal pathways was analyzed. As results shown in Fig. 7, miR-155 mimic transfection increased the expression levels of phosphorylated p38, p65, lκBα, mTOR, and p70S6K, whereas miR-155 inhibitor decreased the expression levels of these factors in LPS-injured cells when compared with inhibitor control group. These findings suggested that RACK1 negatively regulated by miR-155 might decrease the inflammatory injury of cells via deactivating MAPK/NF-κB and mTOR pathways.

## Discussion

The current study showed for the first time that down-regulation of miR-155 protected mouse microglia BV-2 cells against inflammatory injury which was induced by LPS in vitro. Down-regulation of miR-155 significantly decreased the elevated expression levels of pro-inflammatory cytokines (TNF-α, IL-1β, IL-6, and IL-8) in LPS-stimulated BV2 cells. In addition, down-regulation of miR-155 increased cell viability and decreased cell apoptosis of the LPS-injured cells. The mechanistic study showed that these inhibitory effects of miR-155 on BV2 cells were mediated by RACK1 and MAPK/NF-κB and mTOR signaling pathways.

It has been reported that neuro inflammation was triggered by the activation of glial cells [24]. Microglial cells, which comprise of 5 to 12% of brain cells, act as primary effector cells and play a crucial role in neuronal homeostasis, brain’s innate immunity, and neuroinflammatory conditions [25, 26]. Microglial cells could be rapidly activated in response to infection, inflammation, or brain injury, and thus lead to the releases of various inflammatory mediators, including IL-1β, IL-6, TNF-α, nitric oxide, reactive oxygen species, and prostaglandin E2 [24]. It was reported that N9 [27] and BV2 [28] cell lines were often used for in vitro studies about CNS diseases which were involved microglia toll-like receptor signaling. LPS, a bacterial cell wall endotoxin, has been widely used as a potent stimuli which could induce activation of microglia cells. This activation make cells releases cytokines and a host of neurotoxic factors, and thus induce neuronal death [29, 30]. Therefore, BV2 cell line and LPS were selected to build the in vitro model to simulate intracranial infection in the present study. We demonstrated that LPS induced inflammatory injury in BV2 cells via decreasing cell viability, increasing apoptosis, and increasing the expressions and concentrations of pro-inflammatory cytokines (IL-1β, IL-6, IL-8, and TNF-α).

Evidences suggest that miR-155 was involved in many biological processes, including inflammation, immunity and hematopoiesis. MiR-155 has been identified and characterized as a component of primary macrophage, responding to various inflammatory mediators, such as interferon-β, TNF-α, and etc. [31]. It was suggested that the inflammatory mediators up-regulate the expression of miR-155 in macrophages and monocytes [32]. Cardoso et al. demonstrated that bacterial endotoxin LPS could also up-regulate the expression of miR-155 in microglia cells; and miR-155 knockdown significantly decreases the production of nitric oxide, as well as the expression of inflammatory cytokines [33]. Consistent with these previous findings, our present results suggested that LPS induction increased the expression of miR-155 in microglia cells; and down-regulation of miR-155 increased cell viability, decreased apoptosis, as well as the expressions of pro-inflammatory cytokines.

According to the results in the present experiments, we found that RACK1 might be a directly target of miR-155 in BV2 cells, and the expression of RACK1 was negatively regulated by miR-155. RACK1 is a 36-kDa cytosolic protein, which plays a crucial role in regulating cell growth and apoptosis [34, 35]. RACK1 was proved to promote or inhibit cell growth depending on absolute intracellular levels of RACK1 and the type of cells [36]. In our study, knockdown of miR-155 alone increased cell viability, decreased apoptosis and the expression of pro-inflammatory cytokines. However, knockdown of both miR-155 and RACK1 decreased cell viability, increased apoptosis and the expression of pro-inflammatory cytokines. It suggested that down-regulation of miR-155 could not protect microglia cells against inflammatory injury if RACK1 expression was suppressed at the same time. Therefore, RACK1 might play a protective role in LPS-induced inflammatory injury of cells.

We then investigated the underlying mechanisms of which miR-155 down-regulation rescued BV2 cells from LPS-induced inflammatory injury by measuring the activation of MAPK/NF-κB and mTOR signaling pathways in LPS-treated BV2 cells. Specifically, we measured the expression levels of p38 (MAPK protein), p65 and lκBα (NF-κB proteins), and mTOR and p70S6K (mTOR proteins), which are well-known for their pro-inflammatory functions. MAPK proteins mediate essential biological and cellular responses to external stress signals. Moreover, MAPK proteins act as regulators in the synthesis of inflammation mediators. During inflammation, the activity of MAPKs, in particular p38 is increased [37]. NF-κB is considered as a pro-inflammatory signaling pathway as it could increases the expression of pro-inflammatory cytokines, chemokines and adhesion molecules [38]. Yao et al. showed that RACK1 negatively regulates NF-κB activation in response to different stimuli [39]. The in vitro study about microglia cells inflammation proved that NF-κB p50 signaling was involved in regulation effect on inflammatory response of BV2 cells [40]. The mTOR pathway is known to control cell survival, metabolism, and growth [41]. Few mTOR proteins, such as mTOR and p70S6K, were reported to play positive role in inducing inflammation [42]. Consistent with these researchers’ results, the present study also found that miR-155 was related with the activations of MAPK, NF-κB and mTOR signaling pathways, and miR-155 modulated these signaling pathways in a RACK1-dependent manner. Combine with the aforementioned findings of the present study, we conjectured that knockdown of miR-155 protected microglia cells against LPS-induced inflammatory injury via targeting RACK1 and regulating activations of MAPK/NF-κB and mTOR signaling pathways.

## Conclusions

In conclusion, the present study demonstrated that knockdown of miR-155 protected microglia cells from LPS-induced inflammatory injury via targeting RACK1 and modulating MAPK/NF-κB and mTOR signaling pathways. The results in the present study provided the evidence that targeting miR-155 might be a potential therapeutic target for intracranial infection. Further animal and clinical studies are required, which may largely strengthen these findings.

## Abbreviations

CNS: Central nervous system
FBS: Fetal bovine serum
GAPDH: Glyceraldehyde-3-phosphate dehydrogenase
LPS: Lipopolysaccharide
MAPK: Mitogen activated protein kinase
mTOR: Mammalian target of rapamycin
NC: Negative control
NF-κB: Nuclear factor kappa-B
PBS: Phosphate buffered saline
RIA: Radioimmunoassay
TNF-α: Tumor necrosis factor-alpha
