# Correction to: Knockdown of miR-155 protects microglia against LPS-induced inflammatory injury via targeting RACK1: a novel research for intracranial infection

**DOI:** 10.1186/s12950-018-0183-x

**Published:** 2018-04-03

**Authors:** Haiyan Yin, Shuwen Song, Xudong Pan

**Affiliations:** 1 Qingdao University, Qingdao, 266071 China; 2 Department of Neurology, Affiliated Hospital of Jining Medical University, Jining, 272000 China; 3 Department of Infectious Diseases, Jining No.1 People’s Hospital, Jining, 272000 China; 4 Department of Neurology, The Affiliated Hospital of Qingdao University, Qingdao, 266000 China

## Correction

Upon publication of the original article [1] it was highlighted by the authors that the first affiliation of author Haiyan Yin was incorrectly cited in duplicate as “Department of Neurology, Affiliated Hospital of Jining Medical University, Jining, 272000, China”. This should instead read “Qingdao University, Qingdao, 266,071, China”. This error in the author details has since been formally noted in this correction article.
